# Risk factors of exposure to *Aedes albopictus* bites in mainland France using an immunological biomarker

**DOI:** 10.1017/S0950268819001286

**Published:** 2019-07-10

**Authors:** A. Poinsignon, D. Boulanger, F. Binetruy, E. Elguero, F. Darriet, P. Gallian, X. De Lamballerie, R. N. Charrel, F. Remoue

**Affiliations:** 1 MIVEGEC, University of Montpellier, IRD, CNRS, Montpellier, France; 2 Unité des Virus Emergents (UVE), Aix-Marseille University, IRD, Inserm, EHESP, Marseille, France; 3 Etablissement Français du Sang Alpes Méditerranée, Marseille, France

**Keywords:** Arboviruses, ELISA, immuno-epidemiology, vectors, risk of exposure

## Abstract

In recent decades, the invasive *Aedes albopictus* vector has spread across Europe and is responsible for numerous outbreaks of autochthonous arboviral disease. The aim of this study was to identify epidemiological and sociological risk factors related to individual levels of exposure to *Aedes albopictus* bites. A multidisciplinary survey was conducted with volunteer blood donors living in areas either colonised or not by *Aedes albopictus* in mainland France. Individual levels of exposure were evaluated by measuring the IgG level specific to *Aedes albopictus* saliva. The most striking risk factors concerned the localisation and characteristics of the dwelling. Individuals living in areas colonised prior to 2009 or recently colonised (between 2010 and 2012) had higher anti-salivary gland extract IgG levels compared with those who were living in areas not yet colonised by *Ae. albopictus*. The type of dwelling did not seem to impact the level of exposure to *Aedes* bites. People living in apartments had a higher anti-salivary gland extract IgG level than those living in individual houses but the difference was not statistically significant. Interestingly, the presence of air conditioning or window nets was associated with a noticeable reduction in bite intensity.

## Introduction

Globalisation of travel and trade, urbanisation, high population density and climate change represent an increasing risk for the geographic spread of vector-borne diseases including arboviruses that have a major medical impact worldwide. Yellow fever remains the most critical of these diseases with a crude lethality rate between 20% and 60% [1]. Dengue is the most prevalent arboviral disease with about 4 billion people at risk and represents, alone, 84% of the health costs generated by invasive insects [2]. Previously endemic only in South Asia and in Africa, dengue is now present in Europe and in the Americas. The same trend was observed with chikungunya, a particularly disabling pathology originated from Africa, which recently extended to the Caribbean and to the Americas. Within a short time, Zika virus has become a public health concern because of the serious lesions that the virus can cause in the fetus [3].

In recent decades, arboviral diseases have become of public health relevance in Mediterranean regions where the invasive mosquito *Aedes* (*Ae.*) *albopictus* has caused several outbreaks of autochthonous chikungunya and dengue virus infections [4]. The first autochthonous chikungunya epidemic outbreak involving more than 200 confirmed cases (330 possible cases) was reported in summer 2007 in north-eastern Italy [5]. In mainland France, three chikungunya outbreaks occurred in 2010, 2014 and 2017 resulting in 25 cases [6, 7]. Autochthonous dengue outbreaks have also been reported in Europe since 2010; seven in France (2010, 2013, 2014 (three outbreaks), 2015 and 2018) have caused 20 clinically confirmed cases and one in Croatia in 2010 comprising 18 cases. Autochthonous outbreaks have occurred where *Ae. albopictus* is established and when the environmental and/or climatic conditions are suitable for its activity and virus replication (hot season). They followed the new introduction of viruses by travelers returning from areas where the pathogenic agents are endemic.

*Aedes* (*Ae.*) *albopictus*, commonly called the Asian tiger mosquito, is an invasive mosquito species that is rapidly spreading across Europe. Native to South-East Asia, *Ae. albopictus* has been long-established in most parts of southern Europe, with Albania and Italy being the first countries to be colonised in 1979 and 1990, respectively. Today, it is widely established or introduced in 20 European countries [4] and based on risk modelling incorporating climate change projections it has been suggested that over time most of Europe will become more suitable for the establishment of *Ae. albopictus* [8]. In mainland France, the presence of *Ae. albopictus* was first reported in 2004 in the south east of France where it became a considerable nuisance [9]. The rapid spread of *Ae. albopictus* was monitored through an oviposition trap-based surveillance system. The species is currently established in 51 departments and it continues to spread progressively to northern regions of the country. Mainland France is therefore particularly vulnerable to the transmission of tropical arboviruses and thus, the risk of new clusters of local transmission cannot be considered negligible.

The threat of surges of mosquito-borne disease outbreaks will continue since human mobility cannot be reduced. Integrative programs including prevention and outbreak control activities (from vector control to training of medical professionals) should be implemented in areas at risk of transmission to avoid uncontrolled propagation of the pathogens after the confirmation of an autochthonous transmission [10, 11]. To establish appropriate preparedness strategies, the detection and characterisation of human population exposure to the vector are crucial. Classically, the risk of exposure to *Aedes* bites is estimated through the evaluation of *Aedes* mosquito densities which are assessed via entomological techniques such as adult trappings and/or identification of positive breeding sites. These techniques are fastidious, time-consuming and have technical limitations [12]. Moreover, they do not measure the contacts at the human–vector interface and can be applied only at the level of one area/population but do not measure the heterogeneity of individual exposure.

In the past decade, a new immune-epidemiological tool has been developed aimed at evaluating exposure to *Aedes* mosquito bites at the population and individual level. This innovative tool is based on the measure of human IgG responses to salivary proteins of arthropod vectors injected during the bite [13]. As far as the *Aedes* genus is concerned, the tool proved to be a reliable quantitative biomarker of human exposure to specific *Aedes* bites in several environments such as *Ae. aegypti* in Senegal [14], in Bolivia [15] and in Colombia [16], to *Ae. albopictus* in La Reunion island [17, 18], to *Ae. caspius* in the south of France [19] and to *Ae. polynesiensis* in the Pacific [20].

A multidisciplinary survey was conducted in areas either colonised or not by *Ae. albopictus* in mainland France. The serological screening was performed measuring the level of IgG specific to *Ae. albopictus* salivary gland extracts (SGE) in a subsample of this cohort to identify epidemiological and sociological risk factors related to individual levels of exposure to *Ae. albopictus* bites.

## Methods

### Study population

**Fig. 1.**
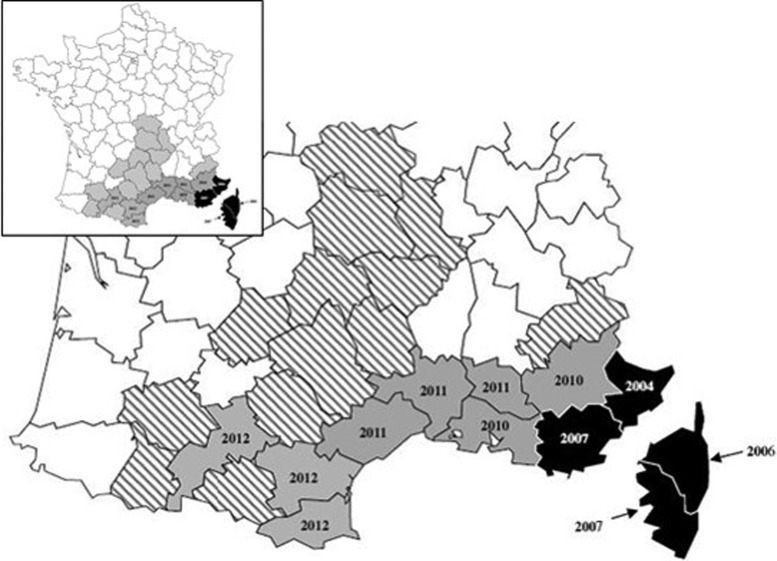
Map of France indicating the year of colonisation by *Aedes albopictus* of studied departments. Volunteer blood donors from 25 departments were included in the survey: four colonised before 2009 (in black), eight colonised between 2010 and 2012 (in grey), 13 not yet colonised at the time of the survey, in 2012 (hatched). Non-concerned departments are void.

The epidemiological design of the study included serum sampling and analysis of epidemiological, environmental, risk perception and behavioural data collected from self-administered questionnaires from individuals residing in mainland France. Individuals were recruited from September to October 2012 during voluntary blood donation in structures of the French Blood Bank located in the departments either colonised or not by *Ae. albopictus* (Fig. 1).

Individuals having travelled in areas where malaria is endemic and in areas where arboviral outbreaks have been reported were deferred 4 months and 4 weeks, respectively, after their return in France. Among the 14 190 people who gave their informed consent for participating in the present study, 9048 returned the sociological questionnaire by either postal service or Internet. From this initial cohort, 246 sera were randomly selected according to the department included in the survey: half from the 12 departments already colonised by *Ae. albopictus* at the time of the survey (on the 17 colonised departments in mainland France) and a half from the 13 non-colonised departments. The main characteristics of the final subsample of the 246 selected individuals are summarised in Table 1. Taking into account the colonisation timetable of the municipality by *Ae. albopictus*, three groups were created: colonisation prior to 2009 (*n* = 59), between 2009 and 2012 (*n* = 30) and not colonised in 2012 (*n* = 157).

**Table 1.** Demographic characteristics of the 246 selected individuals

	Colonised before 2009	Colonised between 2010 and 2012	Not yet colonised in 2012
Number of departments	4	8	13
Number of municipalities	24	21	100
Number of sampled individuals	59	30	157
Sex ratio (M/F)	0.97	1.50	1.29
Age range (years)	21–68	19–66	18–69
Median age	45.0	44.5	48.0
Mean age (95% CI)	44.1 (41–47.2)	41.9 (35.8–47.9)	46.9 (44.5–49.3)
% living in an apartment	45.5	48.3	22.8
% with air conditioning	37.3	27.6	16.6
% with window mosquito screen	37.9	16.7	24.2
% with a garden	55.4	57.1	76.8
% with a terrace/balcony	82.1	82.1	86.2

### Ethics

All procedures relating to the conducting, evaluation and documentation of the study have been conceived in agreement with the good clinical practices and ethical principles of the Helsinki declaration. The protocol was presented to an Ethical Committee (Comité de Protection des Personnes Sud Méditerranée I) and because no additional blood sampling was required out of the blood donation, the committee stated the non-necessity to be consulted. Written informed consent was obtained from all subjects included in the study. The protocol is in agreement with the national regulations on personal data (Commission Nationale Informatique et Liberté). The collection of biological samples was declared to the French Ministry of Research. All data and samples were anonymised.

### Entomology

Routine mosquito surveillance based on a network of oviposition traps was operated by the Établissement Interdépartemental pour la Démoustication (EID) in the framework of the surveillance of the spread of *Ae. albopictus* in metropolitan France. Each ovitrap was checked monthly from June to November 2012. *Ae. albopictus* was considered as established in a municipality when reproduction (visual evidence of *Ae. albopictus* eggs or larvae) and overwintering were evidenced. A department was considered as colonised when *Ae. albopictus* was established in at least one municipality within the department.

### Collection of *Aedes albopictus* salivary gland extracts

SGE were obtained from 10-day-old uninfected female mosquitoes reared in an insectary (IRD, Montpellier, France), as previously described [17]. Briefly, 2 days after a blood meal, the mosquitoes were sedated with CO_2_ and then their salivary glands were dissected out and transferred into a tube containing 30 µl of phosphate buffered saline (PBS) and 5 µl of protease inhibitor (Sigma, St.-Louis, MO, USA). The dissected glands were then pooled in 30 pairs per batch and frozen at −80 °C before extraction. A simple technique consisting of three successive freeze-thaw cycles in liquid nitrogen was used to disrupt the membranes. The soluble SGE fraction was then separated by centrifugation for 20 min at 30 000 ***g*** at +4 °C. The concentration of protein was evaluated by the Bradford method (OZ Biosciences, Marseille, France) after pooling of the different batches to generate a homogenous SGE for immunological assessment. SGEs were then stored at −80 °C before use.

### Evaluation of human IgG Ab levels

An enzyme-linked immunosorbent assay (ELISA) was carried out using Maxisorp plates (Nunc, Roskilde, Denmark) coated with *Ae. albopictus* SGE (2 µg/ml in PBS) at 37 °C for 150 min. Plates were blocked using 250 µl of protein-free Blocking-Buffer (Pierce, ThermoFisher, France) for 60 min at room temperature. Individual sera were incubated in duplicate at a 1/100 dilution in PBS-Tween 1%, 4 °C overnight. Monoclonal mouse biotinylated Ab against human IgG (BD Pharmingen, San Diego, CA) was incubated at a 1/1000 dilution for 90 min at 37 °C. Peroxidase-conjugated streptavidin (GE, Orsay, France) was added at 1/1000 for 60 min at 37 °C. Colorimetric development was carried out using ABTS (2,2′-azino-bis (3-ethylbenzthiazoline 6-sulfonic acid) diammonium) in 50 mM citrate buffer (pH 4) containing 0.003% H_2_O_2_ and absorbance was measured after 120 min at 405 nm. Each serum was assessed in duplicate wells and in a blank well without antigen (OD*n*) to measure non-specific ELISA reactions. Individual results were expressed as the ΔOD value calculated using the equation ΔOD = OD*x*-OD*n*, where OD*x* represents the mean of the OD readings in the two antigen wells.

### Statistical analysis

ΔOD were analysed as quantitative data. Only univariate analyses were performed, the small numbers eligible for inclusion in the study precluding any powerful multivariate analysis. For the sake of clarity among the 64 analysed items, only variables associated with a *P* value <0.15 are detailed. After confirmation of non-Gaussian distributions, non-parametric Mann–Whitney and Kruskal–Wallis tests were used to compare quantitative Ab levels between two independent groups and between more than two groups, respectively. The difference (Δ) in IgG median value between the two groups is indicated in percentage. The Spearman test was used to assess the correlation between IgG levels against *Ae. albopictus* SGE and age. All differences were considered significant at *P* < 0.05.

## Results

### Impact of demographical factors on specific IgG level

There was no clear association between IgG levels specific to *Ae. albopictus* SGE and either sex (*P* = 0.82) or age (*r* < 0.001, *P* = 0.63) of the individuals studied (data not shown).

By contrast, risk factors were mainly environmental, linked to the dwelling representing an interface between vectors and humans.

### Level of exposure according to the biting period

First, we compared the level of IgG anti-SGE according to the time of day during which biting perception was reported. Persons reporting daytime bites (morning, afternoon, or evening) had anti-SGE IgG levels more than twofold higher than those who did not report daytime bites. This difference was statistically significant only when considering afternoon bites (Δ =  + 118%, *P* = 0.028). When considering morning or evening bites, the results were just above the limit for significance (Δ =  + 109%, *P* = 0.056 for morning and Δ =  + 101%, *P* = 0.053 for evening). Individuals reporting night-time bites had a higher, but not statistically significant, level of anti-SGE IgG compared with people reporting no night-time bites (Δ =  + 58%, *P* = 0.387).

### Level of exposure according to year of colonisation of the municipality by *Ae. albopictus*

**Fig. 2.**
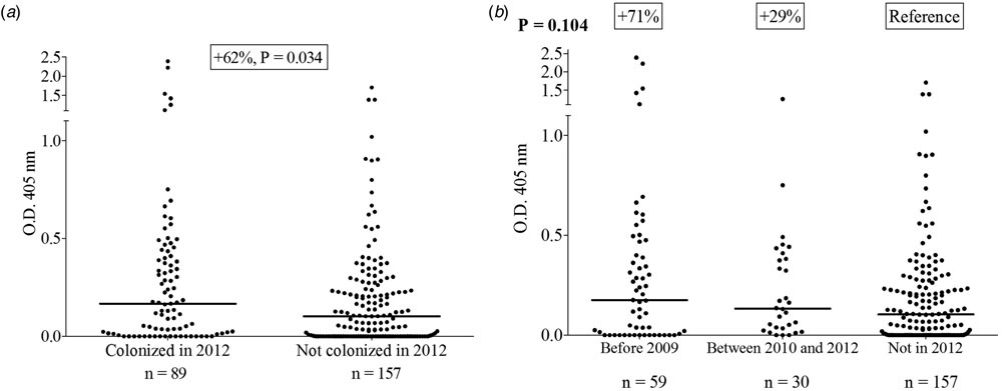
IgG responses to *Ae. albopictus* SGE according to the status colonised *vs.* not colonised in 2012 (a) or to the year of colonisation (b) by *Ae. albopictus* at the scale of municipalities. Each dot presents the individual IgG responses (ΔOD) against *Ae. albopictus* SGE and the horizontal bar indicates the median value. The difference in median value between the two groups is indicated in percentage and the number of processed sera is indicated below (*n*).

We evaluated and compared the level of exposure to *Ae. albopictus* bites, represented by the level of anti-SGE IgG of *Ae. albopictus*, in individuals according to the time of colonisation of the municipality they lived in. The individuals living in municipalities colonised by *Ae. albopictus* during the sampling period (in or prior to 2012) had significantly higher anti-SGE IgG levels than those living in areas not colonised at the time of the sampling (Δ =  + 62%, *P* = 0.034) (Fig. 2a). Although people who were living in areas colonised prior to 2009 or recently colonised (between 2010 and 2012) had higher anti-SGE IgG levels compared with those who were living in areas not yet colonised ( + 71% and + 29% for the median value, respectively), the difference did not reach statistical significance (*P* = 0.07 and *P* = 0.14, respectively), reflecting heterogeneities among individual responses (Fig. 2b). People living in municipalities not colonised by *Ae. albopictus* during the sampling period also had anti-SGE IgG levels with ΔOD values ranging from 0 to 1.7.

### Level of exposure according to the type of dwelling

**Fig. 3.**
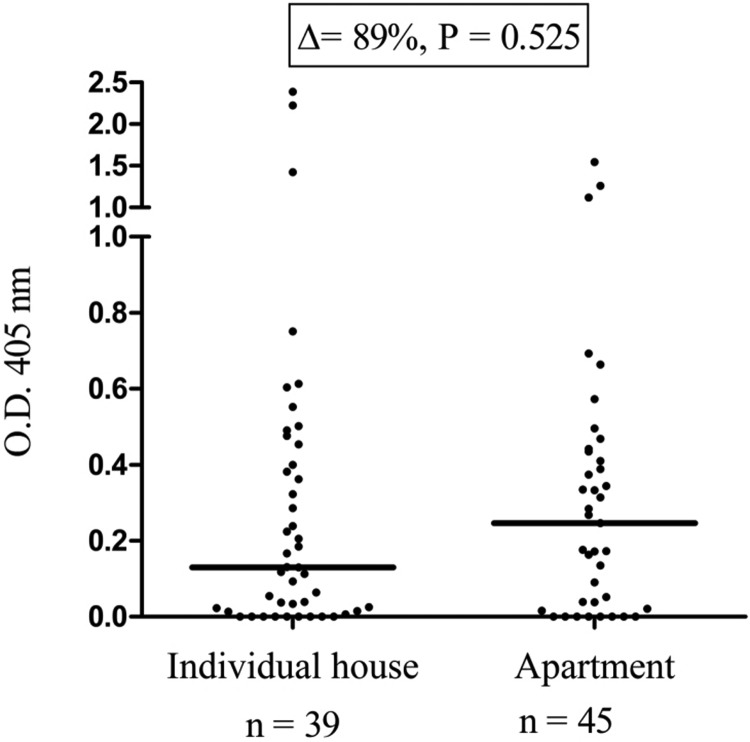
IgG responses to *Ae. albopictus* SGE according to the type of housing. Each dot presents the individual IgG responses (ΔOD) against *Ae. albopictus* SGE and the horizontal bar indicates the median value. The difference in median value between the two groups is indicated in percentage and the number of processed sera is indicated below (*n*).

People from colonised municipalities living in apartments had a higher median anti-SGE IgG level compared with those living in individual houses (Fig. 3, Δ =  + 89%), but this difference was not statistically significant (*P* = 0.525).

### Level of exposure according to physical characteristics of the dwelling

We also investigated the influence of certain physical characteristics of the housing. In municipalities colonised by *Ae. albopictus*, individuals living in housing with mosquito screens on windows had lower anti-SGE IgG levels, but the difference was not statistically significant (Table 2). Only the presence of an air conditioning system drastically decreased anti-SGE IgG levels (Δ = −276%, *P* = 0.022). The presence/absence of a balcony or terrace had no impact, and, surprisingly, the presence of a garden (in 56% of the housing) was linked to a threefold decrease in the median IgG level, but it was not statistically significant. Moreover, the size of the garden did not seem to have a detectable impact (data not shown).

**Table 2.** Influence of domestic equipment on anti-SGE IgG median levels in individuals living in colonised municipalities (*n* = 89)

	Without		With			
Item	% of individuals	IgG median (OD)	% of individuals	IgG median (OD)	Δ (%)	*P*
Air conditioning	65.9	0.222	34.1	0.059	−276.3	**0**.**022**
Window mosquito screen	69.3	0.185	30.7	0.118	−56.8	0.141
Terrace/balcony	17.9	0.185	82.1	0.135	−37.0	0.865
Garden	44.1	0.284	55.9	0.110	−158.2	0.121

Δ (%) represents the difference in the OD value of the median IgG level of individuals with domestic equipment compared with individuals without the item. Bold value denote statistical significance at the *p* < 0.05 level.

## Discussion

Although exposure to *Aedes* bites has already been monitored using anti-saliva antibodies in various epidemiological contexts in tropical regions [13], our study is the first to focus on the risk of exposure to *Ae. albopictus* bites in mainland France. It was designed mainly in response to the increasing pressure created by the rapidly colonising *Ae. albopictus* species and in order to identify potential risk factors of exposure to this vector species. The ability of *Ae. albopictus* to adapt to new environments, its predicted spread and establishment in Europe and its confirmed involvement in disease transmission cycles make the surveillance and control of this species of utmost importance. We were interested in finding out where and how people are bitten by mosquitoes for the dual purpose of (i) improving our knowledge on the basic biological mechanisms of human target recognition by hematophagous flying arthropods and (ii) optimising vector control strategies (VCS) aimed at reducing mosquito–human contacts and thereby potential disease transmission. For an optimum control, the efficacy of VCS must be increased and their environmental side effects must be reduced. The likelihood of being bitten is ruled by both human and mosquito behaviours and can be influenced by environmental factors. Social sciences can provide useful information but they are impeded by human subjectivity in answering questionnaires. The immuno-epidemiological biomarker we have been developing for the past decade has the advantage of providing a validated and quantitative estimate of human exposure to *Aedes* bites [15, 17]. It is based on measuring the level of anti-SGE IgG of *Ae. albopictus* and represents a proxy of human exposure to *Aedes* bites at the individual level. It also has the capacity to discriminate between mosquito species [17, 20]. To identify potential risk factors for *Ae. albopictus* bite exposure, we used this immunological biomarker to screen a well-documented population subsample living in areas with different environmental characteristics within the general framework of the ongoing colonisation by this invasive species. The current data are presented at the municipality level in which the oviposition traps were set up and selected for the routine surveillance of *Ae. albopictus*.

Interestingly, people who reported being bitten by mosquitoes in the morning, afternoon, or evening had significantly higher (i.e. twofold) anti-SGE IgG levels compared with people who reported not being bitten during the daytime. This association confirms that the biomarker of exposure is suitable for evaluating the level of exposure to *Ae. albopictus* bites, as the maximal biting activity of *Ae. albopictus* is during daylight, in agreement with most observations describing bimodal daily feeding activities.

No clear correlation between the risk of *Ae. albopictus* exposure and age groups or gender was found in the present study. Most people tested were in the 40–60 -year age group, limiting the comparison of exposure levels between other age groups. The relationship between age and level of exposure to *Aedes* bites differed according to the epidemiological context. A previous study of anti-saliva IgG responses in Bolivia showed that the intensity of exposure to *Ae. aegypti* decreased with age [15], whereas a similar level of exposure to *Ae. albopictus* bites was observed in urban districts in La Reunion island irrespective of the age group tested in a sample ranging from 18 to 65 years of age [18].

The most striking risk factors concerned the dwellings and in particular their localisation and characteristics. First, the present study showed that people who lived in municipalities colonised by *Ae*. *albopictus* had significantly higher specific anti-SGE IgG than people living in non-colonised places. Interestingly, there was also a positive association between the median value of anti-SGE IgG and the years of colonisation. Individuals living in the municipalities colonised earlier (prior to 2009) had a more intense specific IgG response, Δ = + 32% and Δ = + 71%, than did individuals living in more recent (colonised between 2010 and 2012) or not colonised localities, respectively. People living in municipalities recently colonised (between 2010 and 2012) also had a higher level of anti-SGE IgG (Δ = + 29%) compared with individuals living in areas free of *Ae. albopictus*. There is little doubt that older colonisation will result in higher mosquito densities, hence a higher probability of bites.

Surprisingly, we also observed that people living in areas not yet colonised by the mosquito in 2012 had IgG specific to *Ae. albopictus* SGE. This observation suggests that either people may have moved to colonised areas (for business or personal travel) and/or there are some cross-reactions between *Ae. albopictus* salivary antigens and other hematophagous arthropods. Indeed, one limitation of such a biomarker based on SGE antigens is the possibility that previous exposures to bites in endemic locations for *Aedes* mosquitoes, abroad or in mainland France, might result in detectable serological responses. It must be underlined that individuals returning from areas where malaria is endemic and in areas where arboviral outbreaks have been reported are deferred 4 months and 4 weeks, respectively, after their return in France. Given the short duration of the anti-saliva immunological response (short half-life of specific IgG estimated to be around 2 weeks) [18], the detection of antibodies to insect salivary antigens is unlikely to be due to exposure in tropical areas. By contrast, owing to common salivary proteins between *Culicidae* species [21], cross-reactions with other metropolitan mosquitoes (*Culex* spp.) and particularly *Aedes* species (*Ae. caspius*, *Ae. detritus*) cannot be excluded. Nevertheless, previous studies highlighted a weak cross-reactivity between *Aedes* species [17, 20], suggesting a weak cross-reactivity with more phylogenetically distant blood-feeding species. We can also hypothesise that the colonisation of France by *Ae. albopictus* was minimised. Surveillance of the spread of *Ae. albopictus* is based on the ovitrap system, but because of logistics and cost constraints only a few ovitraps were routinely checked in the departments. This suggests that the entire mainland could not be surveyed and that the surveillance system was certainly not powerful enough to identify all *Ae. albopictus* populations.

The type of dwelling did not seem to impact the exposure to *Ae. albopictus* bites. People living in apartments were bitten more often than those living in individual houses; however, the difference was not statistically significant. Several explanations can be provided, for example, the higher abundance in apartments of domestic sites optimal for *Ae. albopictus* larval breeding such as flower-pot saucers [22]. Alternatively, group housing increases the concentration of physicochemical signals used by mosquitoes to find their targets, such as CO_2_ level and temperature [23].

Unexpectedly, gardens were associated with a threefold decrease in human exposure to *Aedes* mosquitoes. Mosquito breeding sites are water-dependent and one can assume that they are more abundant in surroundings involving high vegetal concentrations. Indeed, land use, vegetation and hydrological characteristics have an impact on mosquito abundance [24]. Some types of gardens have been shown to favour the presence of mosquitoes through the promotion of structural larval habitats such as leaf axils (e.g. Bromeliaceae) [25]. We cannot exclude that individuals owning a garden are more likely to use personal protective repellent such as coils or sprays and are therefore less bitten.

Mosquito nets for windows were also associated with a noticeable reduction in bite intensity but the association did not reach statistical significance. The presence of an air conditioning system in housing significantly reduced the exposure to *Ae. albopictus* bites. In a survey performed across the Texas-Mexico border, the presence of air conditioning was correlated with a lower seroprevalence of dengue virus [26]. Two hypotheses can be proposed: (i) better air tightness mechanically preventing mosquitoes from getting in and/or (ii) the inhibiting effect of cooler temperatures on the level of mosquito feeding activities.

In conclusion, the present study confirmed that people living in areas with high and/or long-standing *Ae. albopictus* densities were more at risk of being exposed to bites and therefore to possible transmission of associated pathogens (mainly arboviruses). We also identified certain risk factors associated with increased exposure to *Ae. albopictus* bites, mainly environmental factors linked to the dwelling characteristics. Use of the immuno-epidemiological biomarker allows for the assessment of individual exposure to *Ae. albopictus* bites and thus the investigation of risk factors that could not be evaluated via classic entomological methods. The recent development of a peptide-based approach proved to be successful in monitoring antibodies directed against specific epitopes present in *Ae. albopictus* salivary proteins [27, 28] and it will be interesting to apply this method in the surveillance of exposure to mosquito bites in mainland France.
